# Differential Expression of microRNAs in Obese Mexican Children: Links to Insulin Resistance and Dyslipidemia

**DOI:** 10.3390/ijms27083396

**Published:** 2026-04-10

**Authors:** Alejandra Contreras-Ramos, Guadalupe Díaz-Rosas, Miguel Cruz, Ana Nava-Cabrera, Miguel Vazquez-Moreno, Omar Gómez-Acuña, Ana María Guerrero-Ortiz, Carmen Domínguez-Hernández, Aleyda Pérez-Herrera, Rosalinda Jiménez-Aguilar, Jaime Goméz-Zamudio, Francisco Javier Gaytán-Cervantes, Miguel Ángel Cid-Soto, Carolina González-Torres, Clara Ortega-Camarillo

**Affiliations:** 1 Laboratorio de Investigación en Biología Molecular, Hospital Infantil de México Federico Gómez (HIMFG), Ciudad de México 06720, Mexico; acontreras@himfg.edu.mx (A.C.-R.); gudiro2@yahoo.com.mx (G.D.-R.);; 2 Unidad de Investigación Médica en Bioquímica, Hospital de Especialidades, Centro Médico Nacional Siglo XXI, Instituto Mexicano del Seguro Social, Ciudad de México 06720, Mexico; 3 OOAD Campeche, Coordinación de Planeación y Enlace Institucional, Instituto Mexicano del Seguro Social, Campeche 24010, Mexico; 4 México SECIHTI, CIIDIR-Unidad Oaxaca, Instituto Politécnico Nacional, Santa Cruz Xoxocotlán 71233, Mexico; 5 Unidad Médica de Alta Especialidad, Hospital General Gaudencio González de la Garza, Centro Médico Nacional “La Raza”, Instituto Mexicano del Seguro Social, Ciudad de México 02990, Mexico; 6 Laboratorio de Secuenciación, División de Desarrollo de la Investigación en Salud, Centro Médico Nacional Siglo XXI, Instituto Mexicano del Seguro Social, Ciudad de México 06720, Mexico

**Keywords:** miRNAs, pancreatic beta cells, insulin resistance, childhood obesity

## Abstract

To analyze, in an analytical cross-sectional observational study, the relationship between the plasma microRNA (miRNA) expression profile in children living with obesity and their metabolic health status. Based on body mass index percentiles (BMIp), the children were grouped into a control group (C) or an obesity group (Ob). Glucose, insulin, and low- and high-density lipoproteins (LDLs and HDLs, respectively), triacylglycerols (TG), and total cholesterol (TC) were measured. RNA from plasma was used for miRNA sequencing analysis (NextSeq 2000 platform). Differential miRNA expression was determined using counts obtained from the reference genome. Fifty controls (BMIp: 50.4 ± 23) and fifty children with obesity (BMIp: 97.54 ± 1.46) were included. The obese group presented hyperinsulinemia and insulin resistance. Sequencing revealed nine underexpressed and six overexpressed miRNAs in the obese group. In silico analysis suggested that these miRNAs may participate in regulating insulin secretion, protein synthesis, apoptosis, and the glycolytic pathway in pancreatic β-cells. Childhood obesity was associated with altered circulating levels of microRNAs linked to glucose metabolism, insulin resistance (IR) and β-cell survival. Reduced plasma levels of miR-126-3p, let-7a-5p, and miR-16-5p showed a high predictive value for hypertriglyceridemia and insulin resistance, indicating their potential relevance as early biomarkers or therapeutic targets in pediatric metabolic dysfunction.

## 1. Introduction

The high palatability of carbohydrate- and fat-rich foods encourages their overconsumption, which significantly contributes to excessive weight gain from an early age. Obesity is a global epidemic that is closely linked to the development of chronic metabolic diseases. According to recent estimates from the World Health Organization, approximately 35 million children under the age of five were overweight by 2024, with a notable increase in low- and middle-income countries [1].

In the Mexican context, the situation is particularly alarming: 35.6% of children between 5 and 11 years old and 38.4% of adolescents between 12 and 19 years old live with obesity [2]. Obesity has immediate effects on quality of life and is associated with a significant predisposition to the premature development of chronic diseases such as metabolic syndrome, cardiovascular diseases and type 2 diabetes (T2D) [2]. Central adiposity plays a key role in the development of these diseases. Excess visceral fat promotes low-grade chronic inflammation and impairs insulin signaling, leading to insulin resistance (IR) [3]. In addition, elevated triglyceride levels together with reduced high-density lipoprotein (HDL) cholesterol, an atherogenic lipid profile, further accelerate the progression toward dysglycemia and T2D [4]. In this same context, experimental models of obesity have shown that this condition can induce a progressive loss of pancreatic β-cells even in the absence of hyperglycemia [5]. This finding indicates that both β-cell mass and function begin to deteriorate at early stages, before the disease becomes clinically detectable. Together, these processes reflect the complex interplay between obesity, metabolic dysfunction, and β-cell impairment, which has stimulated growing interest in elucidating the molecular mechanisms underlying these alterations. Additionally, the role of epigenetic factors, such as microRNAs (miRNAs), has emerged as a critical aspect in understanding the pathophysiology of obesity and T2D [6]. These small posttranscriptional regulators participate in multiple cellular processes, and it has been suggested that alterations in their expression profile in overweight or obese individuals could be early indicators of the development of metabolic diseases. Therefore, changes in the miRNA expression profile in overweight or obese individuals may indicate the development of metabolic diseases. In adults with obesity, specific miRNAs—such as miR-17-5p, miR-132, miR-99a, miR-134, miR-181a, miR-145, and miR-197—have been associated with visceral fat accumulation; the percentage of Glycated hemoglobin (HbA1c), glycemia, leptin, adiponectin and interleukin 6, parameters that predispose to metabolic syndrome and T2D [7]. Likewise, reduced miR-126 expression or increased levels of miR-27a, miR-320, and miR-375 have been proposed as potential biomarkers of T2D. In pediatric populations, differences in miRNA expression have been linked to preadipocyte proliferation, glucose intolerance, and reduced insulin secretion, suggesting that miRNAs could be used as predictive markers for the development of metabolic disorders in obese children [8]. Consistently, several miRNAs—including miR-551a, miR-501-5p, miR-10b-5p, miR-191-3p, miR-215-5p, miR-874-3p, miR-221, miR-28-3p, miR-142-3p, miR-486-5p, and miR-486-3p—have been associated with childhood obesity, its severity, and the risk of future metabolic comorbidities [6]. The objective of this cross-sectional case–control study was to evaluate miRNA expression patterns associated with obesity and insulin resistance and to examine their relationship with early metabolic indicators, with the goal of identifying epigenetic biomarkers that reflect the onset of metabolic impairment. MicroRNA expression data could be used to develop preventive or intervention programs, as it can identify early molecular alterations associated with metabolic risk, particularly in obese children and adolescents.

## 2. Results

### 2.1. Characteristics of the Participants

Children were assigned based on their body mass index percentile (pBMI) to the control group (C) or the obesity group (Ob). General and anthropometric characteristics are shown in Table 1. Forty percent of the participants were girls in both groups. No differences were observed in height, age, or blood pressure. The pBMI and waist circumference were greater in the Ob group (*p* < 0.001). And then, children with obesity showed markedly higher central adiposity compared with their normal-weight peers. The waist-to-height ratio (WHtR) was significantly elevated in the obesity group (0.60 ± 0.06) relative to normal-weight children (0.46 ± 0.04) (Table 1). With respect to the metabolic data, hyperinsulinemia and insulin resistance were significantly greater in the obese group than in the control group. Additionally, the cardiometabolic index (CMI) was higher in obese children (1.86 ± 1.05) than in normal-weight children (1.17 ± 0.84). This finding suggests a consistent increase in cardiometabolic burden among obese children (*p* < 0.05). HOMA-β estimates revealed that children living with obesity also exhibited higher pancreatic beta cell functional activity than their normal-weight peers (Table 2, *p* < 0.001).

### 2.2. Profile of miRNAs in Plasma from Normal Weight Children and Children Living with Obesity

**Figure 1:**
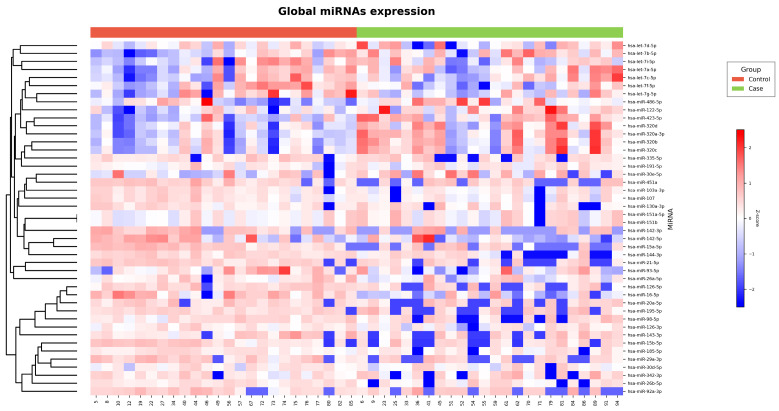
Plasma miRNA sequencing analysis in children with obesity (n = 25) and controls (n = 25). Heatmap showing Z-score–normalized expression levels of miRNAs (red, higher expression; blue, lower expression). Samples are grouped by experimental condition, with controls shown in red and cases in green. miRNAs were hierarchically clustered according to their expression profiles.

**Figure 2:**
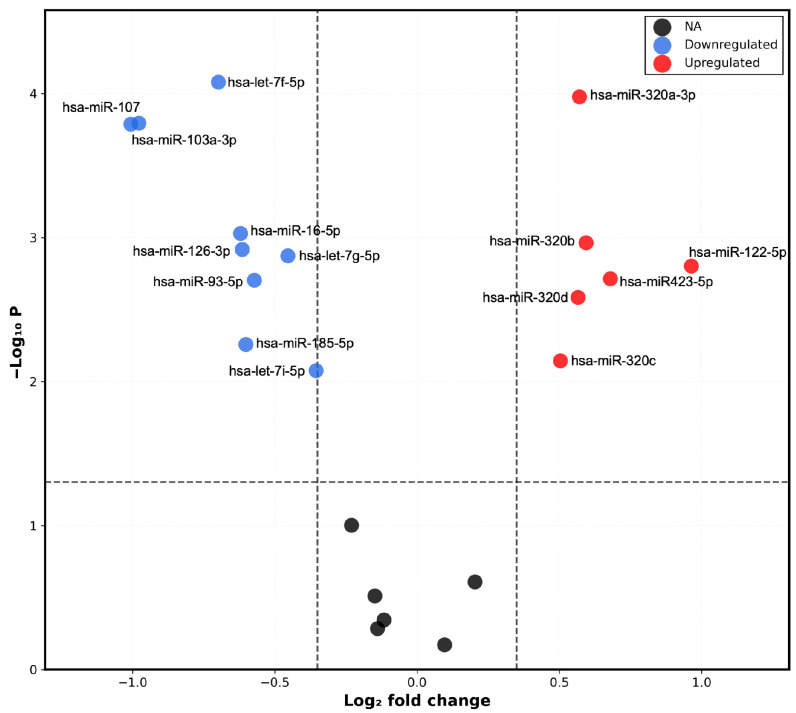
Differential expression of plasma miRNAs in children with obesity and normal-weight controls. Volcano plot of differentially expressed miRNAs, showing log_2_ fold change (logFC) on the *X*-axis and −log_10_(*p*-value) on the *Y*-axis. Significantly downregulated miRNAs are highlighted in blue, and significantly upregulated miRNAs in red. Statistical significance was defined as |log_2_FC| ≥ 0.5 and FDR < 0.05 (dashed lines).

The results of the sequencing study were visualized in a heatmap (Figure 1) and a volcano plot (Figure 2). A differential microRNA expression profile was observed in the plasma of children with obesity versus controls. Nine downregulated miRNAs (hsa-let-7f-5p; hsa-miR-107; hsa-miR-103a-3p; hsa-miR-16-5p, hsa-miR-126-3p; hsa-let-7g-5p; hsa-miR-93-5p; hsa-let-7i-5p; hsa-miR-185-5p) and six upregulated miRNAs (hsa-miR-320a-3p; hsa-miR-320-3b; hsa-miR-122-5p; hsa-miR-423-5p; hsa-miR-320d; hsa-miR-320c) were identified in the Ob group.

### 2.3. In Silico Analysis of Overexpressed miRNAs in Plasma from Control Children and Children Living with Obesity as Candidates Associated with Obesity and Pancreatic β-Cell Dysfunction

**Figure 3:**
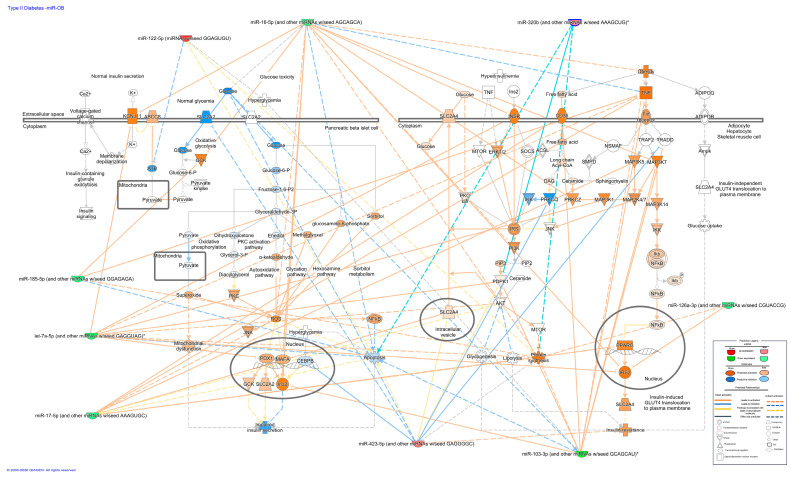
miRNA–mRNA interaction network generated using Ingenuity Pathway Analysis (IPA^®^, QIAGEN), integrating predicted microRNA targets within the Type 2 diabetes signaling pathway across β-cells, adipocytes, hepatocytes, and skeletal muscle. The network highlights key metabolic regulators and potential post-transcriptional control points contributing to insulin resistance and glucose dysregulation. * Nucleotides in parentheses indicate the conserved seed sequence (for example, GAGGTA) of the canonical microRNA recognition motif essential for target binding.

**Figure 4:**
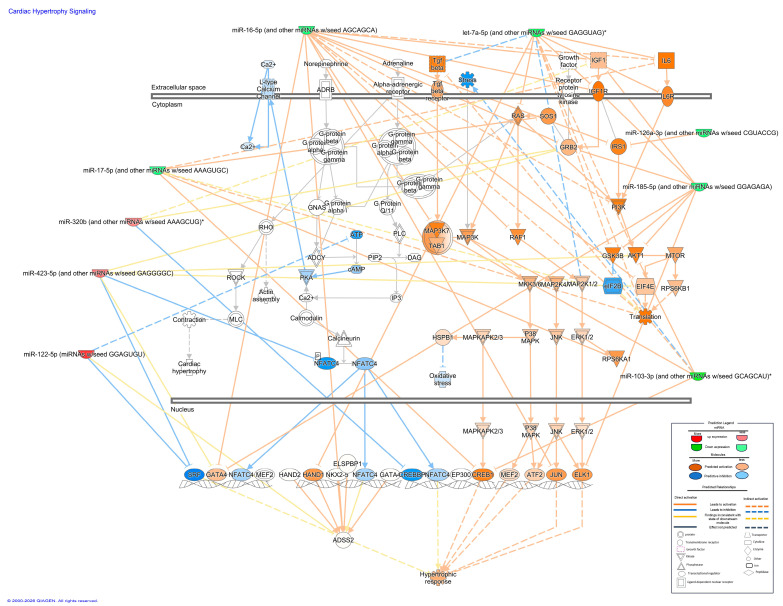
IPA^®^-derived miRNA–mRNA network illustrating the predicted post-transcriptional regulation of Cardiac hypertrophy-related genes by candidate microRNAs. * The canonical microRNA recognition motif’s conserved seed sequence is shown by the nucleotides in brackets.

The pathway analysis suggested that the miRNAs identified by sequencing were associated with metabolic and stress-response networks relevant to type 2 diabetes, pancreatic β-cell apoptosis, and cardiac injury (Figure 3 and Figure 4). Using Qiagen’s IPA software (Version 01-24), we observed that the microRNAs (miRNAs) identified by RNA sequencing (RNAseq) (let-7f-5p, hsa-mir-107, hsa-mir-103a-3p, hsa-mir-16-5p, hsa-mir-126-3p, hsa-let-7g-5p, hsa-mir-93-5p, hsa-let-7i-5p, hsa-mir-185-5p) and six upregulated miRNAs (hsa-mir-320a-3p, hsa-mir-320-3b, hsa-mir-122-5p, hsa-mir-423-5p, hsa-mir-320d, hsa-mir-320c) participate in signaling cascades involved in glucose metabolism, insulin secretion, and β-cell survival. They were also predicted to participate in pathways associated with cardiovascular damage and the induction of genes linked to cardiac remodeling, which is a characteristic of dilated and hypertrophic cardiomyopathy. Within this profile, miR-126-3p, miR-16-5p, and let-7a-5p showed the strongest predicted connectivity to genes participating in these processes. Their putative targets clustered in nodes related to glucose handling and apoptotic signaling in β-cells, while also intersecting with pathways associated with increased cardiac risk.

The analysis of three members of the let-7 family (let-7i, let-7f, and let-7g) in IPA showed that their predicted targets converged on a shared regulatory module. To visualize this overlap, the network was clustered around let-7a, the most representative family member, which contains the conserved seed sequence GAGGTA. This approach allowed the construction of a unified node highlighting the common downstream genes and pathways modulated across the let-7 family. Based on this convergence, let-7a-5p was selected for expression validation by qPCR in plasma samples.

### 2.4. Expression of miRNAs and Their Target Genes in the Plasma of Control Children and Children Living with Obesity

In this part of the study, we evaluated the expression of miRNAs in plasma, focusing on miR-126-3p, Let-7a-5p and miR-16-3p. These miRNAs were selected based on previous reports that have highlighted their involvement in metabolic and cardiovascular pathways that are relevant to paediatric obesity and related diseases. MicroRNA-126-3p (miR-126-3p) is a well-established regulator of endothelial function and insulin sensitivity. Reduced circulating levels of this miRNA have been associated with obesity and the early onset of cardiometabolic abnormalities in children [9,10]. Let-7a-5p, a member of the Let-7 family, has been consistently associated with glucose metabolism, insulin signalling and beta cell function. Its increased expression has been reported in paediatric obesity and has been found to correlate with insulin resistance [10]. MicroRNA-16-3p (miR-16-3p) is involved in pathways related to inflammation, glucose homeostasis and insulin signalling. Altered levels of this miRNA have been observed in individuals with metabolic dysfunction, including obese children [11].

#### 2.4.1. Expression of miRNA-126-3p and Its Target Genes

**Figure 5:**
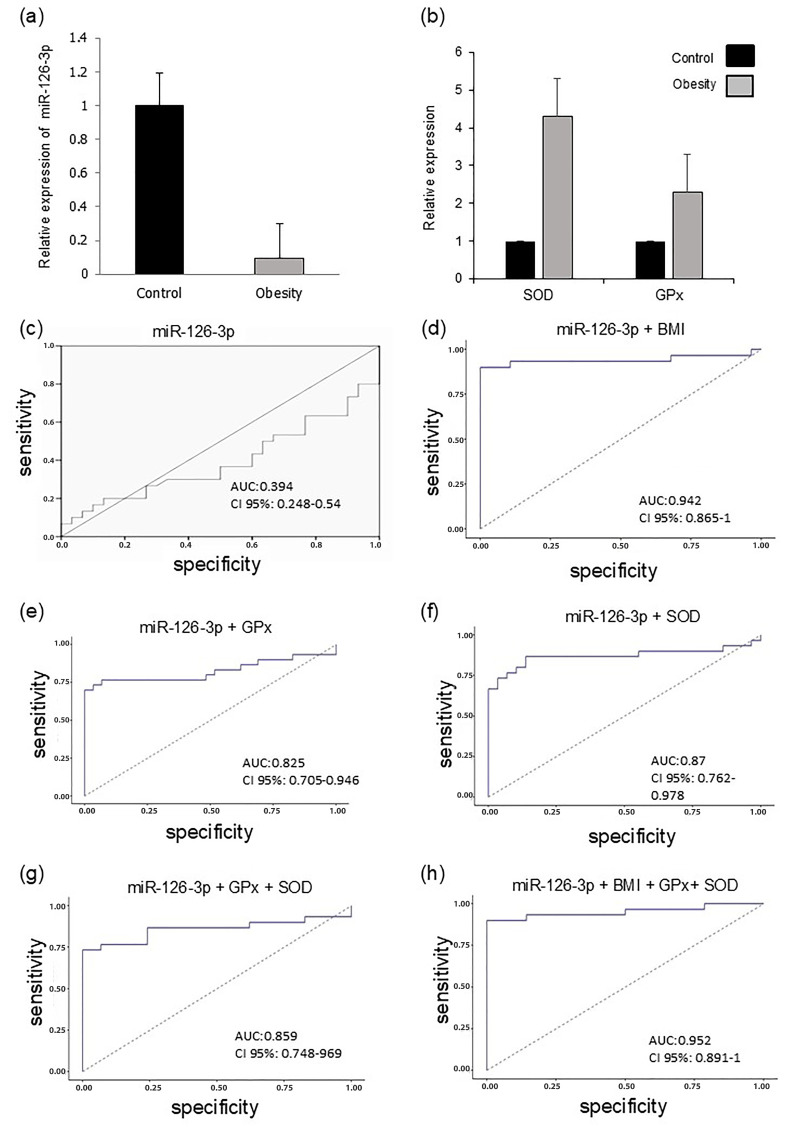
(**a**) Relative expression of miR-126-3p. (**b**) Superoxide dismutase (*SOD*) and glutathione peroxidase (*GPx*) in the plasma of children living with obesity and normal-weight children. (**c**) ROC (receptor operating characteristic) curve analysis of plasma miR-126-3p levels in obese patients vs. control. The sum of the logarithms of plasma miR-126-3p levels with (**d**) pBMI, (**e**) *GPx*, (**f**) *SOD*, (**g**) *GPx* + *SOD*, and (**h**) pBMI + *GPx* + *SOD* was included for comparison.

Quantitative PCR analysis confirmed the sequencing results and showed a significant decrease in miR-126-3p expression in children living with obesity compared with the normal-weight group (Figure 5a).

Because obesity is known to induce oxidative stress and disrupt antioxidant defenses, we examined the gene expression of the antioxidant enzymes superoxide dismutase (*SOD*) and glutathione peroxidase (*GPx*), which are two Nrf2-regulated enzymes involved in the detoxification of reactive oxygen species [12]. The results revealed marked increases in *SOD* and *GPx* expression levels, which were approximately four-fold and two-fold higher, respectively, in children living with obesity relative to normal-weight participants (Figure 5b).

Pearson correlation analysis revealed a significant association between decreased miR-126-3p expression and pBMI (r = −0.396; *p* < 0.05) and *GPx* (r = 0.897; *p* < 0.001). *SOD* antioxidant enzyme expression correlated positively with total lipid levels (r = 0.617; *p* < 0.01), cholesterol levels (r = 0.581; *p* < 0.01), triacylglycerol levels (r = 0.415; *p* < 0.001), and LDL levels (r = −0.746; *p* < 0.01). For prediction, miR-126-3p was used for logistic regression analysis (AUC: 0.394; 95% CI: 0.248–0.54; Figure 5c). The diagnostic capacity of the model increased significantly when the pBMI and the antioxidant enzymes *SOD* and *GPx* were integrated (Figure 5d–f). ROC curve analysis revealed that the AUC increased to 94% when combining miR-126-3p with pBMI (95% CI: 0.865–1; *p* < 0.001), to 82% with *GPx* (95% CI: 0.705–0.969; *p* < 0.001), and to 85% with *SOD* (95% CI: 0.748–0.969; *p* = 0.036). Notably, the triple combination of miR-126-3p, *GPx*, and *SOD* achieved an AUC of 87% (95% CI: 0.762–0.978; *p* = 0.028; Figure 5g). Optimal performance was obtained using a multivariate model that integrated all four parameters (miR-126-3p, pBMI, *GPx*, and *SOD*), achieving an AUC of 97% (95% CI: 0.891–1; Figure 5h). These findings suggest that this molecular and enzymatic signature may serve as a useful indicator of metabolic stress in childhood obesity.

#### 2.4.2. Let-7a-5p Expression

**Figure 6:**
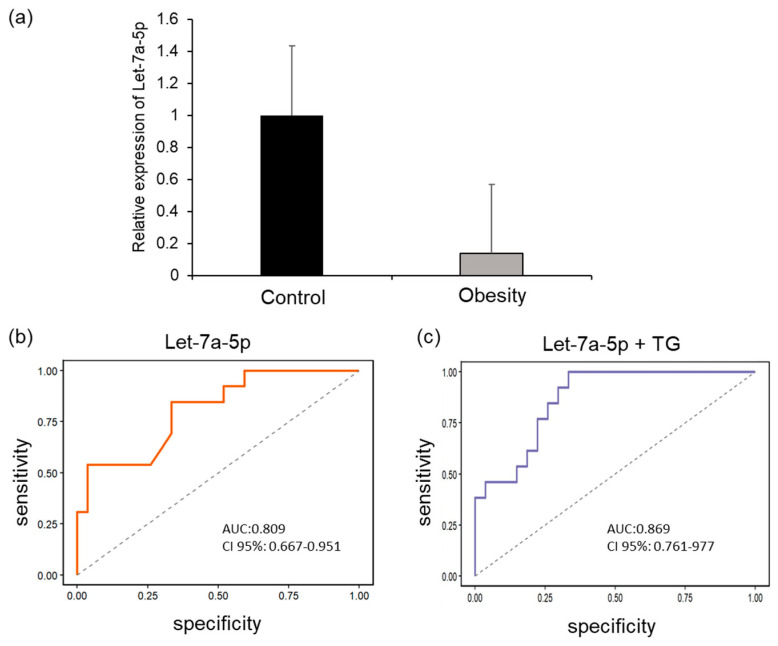
Relative expression of let-7a-5p. (**a**) let-7a-5p expression in the plasma of children living with obesity and normal-weight children. (**b**) ROC curve analysis of plasma levels of let-7a-5p in obese patients vs. control. (**c**) Additionally, the sum of the logarithms of plasma levels of let-7a-5p with Triacylglycerides (TG) was analyzed.

Let-7a-5p expression levels, determined by qPCR of plasma samples from children living with obesity, were significantly lower compared with those of children of normal weight. These data confirm the findings of the sequencing study (Figure 6a). Let-7a-5p expression correlated with triacylglycerol levels (r = −0.454, *p* < 0.05). Regarding its diagnostic capacity, let-7a-5p showed an AUC of 80% (95% CI: 0.667–0.951, *p* < 0.001; Figure 6b) for identifying obesity. Notably, this accuracy improved when triacylglyceride concentration was included in the model, reaching an AUC of 86% (95% CI: 0.761–0.977, *p* = 0.003; Figure 6c). These results suggest that the combined panel of let-7a-5p and triacylglycerides may provide a more informative indicator of metabolic risk in childhood obesity.

#### 2.4.3. miR-16-5p Expression

**Figure 7:**
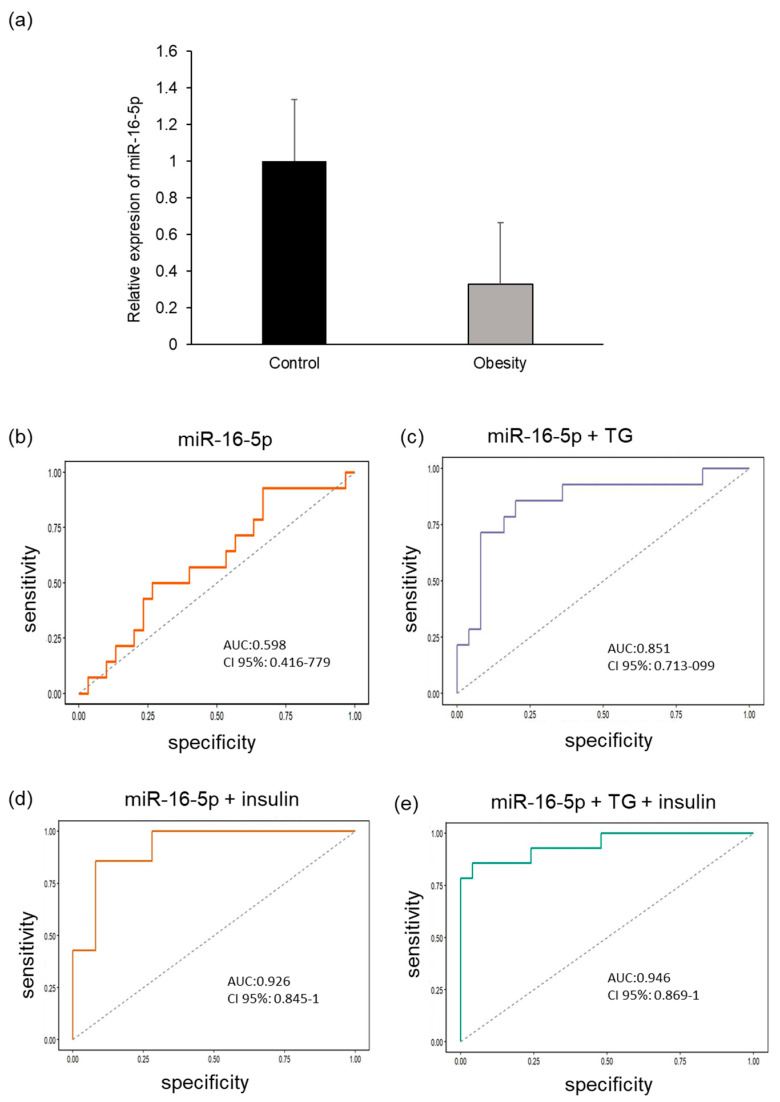
(**a**) Relative expression of miR-16-5p in the plasma of children living with obesity and normal-weight children. (**b**) ROC curves for the predictive value of miR-16-5p in obesity. The diagnostic efficacy of miR-16-5p in patients with obesity is potentiated by (**c**) triacylglycerides, (**d**) insulin, and (**e**) triacylglycerides and insulin.

In terms of miR-16-5p expression, a slight decrease in its plasma expression was observed in children with obesity compared with that in children with normal weight; however, the *p*-value was not significant (Figure 7a). Therefore, these results support those of previous sequencing analyses (Figure 1 and Figure 2).

Although miR-16-5p showed limited diagnostic capability in isolation, with an AUC of 0.59 (95% CI: 0.416–0.779; Figure 7b), its correlation analysis of Pearson revealed a significant association with key metabolic parameters. Specifically, the reduction in miR-16-5p was strongly correlated with insulin concentration (r = −0.586, *p* < 0.01) and HOMA-IR (r = −0.582; *p* < 0.01) and also showed links with triacylglycerol (r = −0.582, *p* < 0.05). Notably, the diagnostic performance of this miRNA was significantly increased when combined with other biomarkers. The AUC increased to 0.85 with the addition of triacylglycerides and to 0.92 with insulin. Finally, the multivariate model integrating miR-16-5p, triacylglycerides, and insulin achieved an optimal diagnostic accuracy of 94% (AUC 0.94; 95% CI: 0.869–1, *p* = 0.01; Figure 7e).

## 3. Discussion

The biochemical and anthropometric parameters of the children included in this study show an increase in the lipid profile, as well as in the HOMA-IR, HOMA-β, and CMI indices. These findings are consistent with previous research on metabolic changes and the higher risk of cardiovascular disease, metabolic syndrome, and T2D in children with obesity. The increases between normal-weight and children with obesity in WHtR indicate a higher adiposity grade and alterations in the lipid profile [13], which contributed to a significantly higher CMI [14,15]. In addition, insulin-related markers showed marked differences between groups. Furthermore, the increase in HOMA-IR and HOMA-β in obese children suggests greater alterations in insulin-related metabolic processes and increased activity of pancreatic beta cells. The clustering of elevated WHtR, CMI, HOMA-IR, and HOMA-β aligns with previous evidence showing that excess adiposity and unfavorable lipid ratios tend to co-occur with alterations in insulin dynamics during childhood. These findings align with evidence indicating that childhood obesity is a metabolic state that leads to premature changes in lipid transport, insulin resistance, and impaired lipoprotein metabolism [13,16,17].

Clarifying the mechanisms that activate obesity and its consequences for metabolic health is a significant challenge, especially in infants. In recent years, changes in microRNA expression have been associated with the presence of metabolic disorders [18], including obesity in adults [19] and children [20].

In this study, we analyzed the changes in the expression profiles of miRNAs in the plasma of children living with obesity versus control children using next-generation sequencing with the NextSeq 2000 platform. Among children living with obesity, nine underexpressed miRNAs were identified (hsa-let-7f-5p, hsa-miR-107, hsa-miR-103a-3p, hsa-miR-16-5p, hsa-miR-126-3p, hsa-let-7g-5p, hsa-miR-93-5p, hsa-let-7i-5p, and hsa-miR-185-5p), as were six overexpressed miRNAs (hsa-miR-320a-3p, hsa-miR-320-3b, hsa-miR-122-5p, hsa-miR-423-5p, hsa-miR-320d, and hsa-miR-320c). These results differ from those for miRNAs previously reported in samples from overweight or mildly obese children (increased expression levels of miR551a and miR-501-5p and decreased expression levels of miR-10b-5p, miR-191-3p, miR-215-5p and miR-874-3p) [8]. In addition, biomarkers of the risk and degree of childhood obesity (miR-221, miR-28-3p, miR-142-3p, miR-486-5p and miR-486-3p) have been proposed [21]. The levels of the miRNAs -126-3p, -576-5p, -10b-5p and -31-5p were reported to be associated with the level of C-reactive protein [22]. The discrepancies observed in the miRNA profiles may be due to various factors, such as the study population and the technology used for their detection [23]. Previously reported miRNAs were identified in populations of European origin [8], and Asians and African Americans, highlighting the differences in miRNA expression associated with ethnic and genetic characteristics [24]. In this study, which was conducted in a Mexican population, the differences found in the miRNA profile could be related to the genetic, environmental and sociocultural characteristics of this population, which highlights the importance of considering the population context in the interpretation of transcriptomic data.

The miRNAs identified in this study were predicted (in silico analysis) to be associated with pathways related to glucose metabolism, proliferation, and apoptosis in pancreatic β cells and were therefore selected for evaluation by qPCR. The decreases in the expression levels of miR-126-3p, miR-16-5p and let-7a-5p observed by PCR confirmed the sequencing results. Our results concerning miR-126-3p expression are consistent with reports from the TODAY cohort study in adolescents, where decreased miRNA-126-3p expression was negatively associated with pancreatic beta-cell function as measured by the C-peptide disposition index [25]. However, other authors reported that increased levels of miR-126-3p in adolescents with obesity [9,26] and children with obesity aged 10–12 years were associated with BMI [27], the development of insulin resistance and the HOMA-IR index [10]. Owing to its extensive involvement in the regulation of transcription factors in endothelial cells and in the regulation of angiogenesis, miR-126-3p is part of the angio-miRs group [28] and is associated with endothelial dysfunction in patients with diabetes [29].

MicroRNAs are associated with the presence of oxidative stress under various circumstances. Obesity is characterized by a state of low-grade inflammation and the presence of oxidative stress. In this study, children living with obesity had elevated levels of the antioxidant enzymes SOD and GPx. Decreased levels of miR-126-3p, along with marked oxidative damage and elevated levels of miR-21 and GPx, have been proposed as biomarkers of cardiovascular complications in adult patients with type 2 diabetes [29]. In the case of miR-126-3p, it has been shown to decrease oxidative stress by promoting Nrf2 expression in renal ischemia [12,30]

The expression levels of let-7a-5p and miR-16-5p were also low in the plasma of children with obesity. The decrease in miR-16-5p correlated positively with insulin, HOMA-IR, and triacylglycerols levels. The expression of let-7a-5p correlated only with the triacylglycerol level.

Experimental evidence shows that let-7a-5p downregulation increases lipid accumulation by activating SREBF2 and PI3K–Akt–mTOR axis, thereby promoting adipocyte expansion and systemic lipotoxicity [31]. These findings are in accordance with a role for let-7a-5p as a negative regulator of fatty-acid storage, positioning it as a protective factor against excessive lipid deposition and its metabolic consequences. This is consistent with the KEGG pathway enrichment results, in which let-7a-5p showed significant representation within the Wnt signaling pathway (FDR = 0.0456), a central regulator of cardiac remodeling, hypertrophic growth, and extracellular matrix dynamics. However, our data contradicts that published by Krause B.J. et al., who reported increased levels of let-7a-5p in children with obesity who also presented three characteristics of metabolic syndrome [10]. Nevertheless, these authors propose that let-7 increases as a biomarker of metabolic syndrome in children with obesity due to its positive correlation with the HOMA-IR index [10,31].

The convergence of let-7a-5p targets in Wnt-related components further supports its functional involvement in structural and metabolic alterations associated with cardiomyopathy [32]. These observations suggest that a reduction in this microRNA may favor visceral fat accumulation, a well-recognized cardiovascular risk factor [33]. Further supporting its involvement in metabolic and structural alterations linked to cardiometabolic disease, let-7a-5p also showed functional clustering of its target genes toward KEGG pathways directly implicated in cardiac remodeling. Its predicted targets were significantly enriched in the PI3K–Akt and MAPK signaling pathways (FDR < 0.05), both of which have been associated with hypertrophic growth, extracellular matrix remodeling, and cardiomyocyte [34]. Therefore, reduced let-7a expression observed in obesity has been proposed to shift the balance between the PI3K-Akt and MAPK pathways toward MAPK-dominant signaling a pattern linked to hypertrophy, fibrosis, and increased cardiovascular risk.

Regarding the expression of miR-16-5p, a reduction (~30%) in its expression has also been demonstrated in children with obesity, and strongly correlated negatively with insulin, triacylglycerol and HOMA-IR levels, supporting the idea that the reduction in this microRNA is an early event in the metabolic dysregulation preceding the onset of T2D. Consistent with our results, studies in humans show that lower miR-16-5p levels are associated with impaired insulin signaling in individuals with T2D or impaired glucose tolerance [35]. Experimental in vitro models further demonstrate that loss of miR-16-5p in skeletal muscle reduces insulin sensitivity [11], whereas its overexpression enhances glucose uptake in myocytes [36]. Together, these findings indicate that reduced miR-16-5p expression may be sufficient to induce insulin resistance [11]. In line with this evidence, impaired muscle function and glucose intolerance are commonly observed in individuals with T2D and obesity. These findings suggest that a lack of miR-16-5p during childhood obesity could be linked to sarcopenia in adults with obesity and T2D. Furthermore, it is known that miR-16-5p expression is regulated by caloric intake [36]. Weight gain leads to a state of chronic inflammation, and calorie-restricted mice have increased expression of miR-16-5p, which inhibits the expression of inflammatory cytokines [37]. Therefore, the decrease in miR-16-5p in the obesity group may be a contributing factor to the inflammation seen in patients with obesity.

Based on the results obtained, the decreases in the levels of miR-126-3p, let-7a-5p and miR-16-5p in the plasma of children with obesity showed a high predictive value for the presence of hypertriglyceridemia and insulin resistance. These findings suggest that these microRNAs may serve as potential epigenetic indicators of early metabolic alterations in pediatric populations with obesity.

### Study Limitations

A major limitation of this study is that it was conducted in only one hospital. This restriction may reduce the representativeness of the study population and limit the generalizability of the findings. Therefore, the results should be interpreted with caution, as they may not fully reflect the diversity of sociodemographic and clinical characteristics present in other populations.

## 4. Materials and Methods

### 4.1. Sample Collection

A total of 100 children between the ages of 6 and 12 were randomly selected to participate in this analytical cross-sectional study from the Primary Care Unit 23 of the Mexican Social Security Institute (IMSS) in Mexico City. Parents signed informed consent forms, and the children gave their assent. The research ethics committee and the National Scientific Research Committee of the IMSS reviewed and approved the study (R-2020-785-131). Anthropometric measurements and physiological parameters (weight, height, waist circumference, hip circumference, and blood pressure) were recorded in the presence of the parents. Blood samples were obtained after at least 6 h of fasting. Serum was separated for the measurement of glucose, insulin, triacylglycerols, total cholesterol, and low- and high-density lipoproteins (LDL and HDL, respectively). RNA was extracted from the plasma samples for sequencing analysis.

### 4.2. HOMA-IR and HOMA-β Index

The fasting glucose and insulin concentrations (insulin concentration determined by ELISA with the 80-INSHU-E01.1 (ALPCO) kit), the insulin resistance index (HOMA-IR = (fasting glucose [mmol/L] × fasting insulin [µU/mL])/22.5) and pancreatic β-cell functionality (HOMA-β = (20 × fasting insulin [µU/mL])/(fasting glucose [mmol/L] − 3.5)) were used.

### 4.3. Cardiometabolic Index (ICM)

The cardiometabolic index (CMI) was calculated using waist circumference, height, and the HDL cholesterol/triglyceride ratio (mg/dL). Waist circumference was measured by a trained professional using a non-elastic measuring tape, and height was measured using a calibrated stadiometer. The CMI was calculated with the following formula: (TG/HDL-C) × (WC/Height) [14].

### 4.4. Total RNA Extraction, miRNAs and cDNA Synthesis

Total RNA and miRNA extraction were performed using an miRNeasy Serum/Plasma Advanced Kit (Qiagen, Germantown, MD, USA), RNeasy MinElute Spin Columns, Collection Tubes (1.5 mL and 2 mL) and QIAzol lysis reagent, RNase-Free Reagents and Buffers (Qiagen Cat No./ID: 217204, Germantown, MD, USA). cDNA synthesis was performed using a miRCURY LNA RT kit (Qiagen Cat No./ID: 339340) following the manufacturer’s instructions.

### 4.5. MicroRNA Expression Profiling and Differential Expression Analysis

The miRNA sequencing libraries were created using next-generation sequencing (NGS) from 5 µL RNA aliquots extracted from plasma/serum samples using QIAseq miRNA library kits (QIAGEN, Germantown, MD, USA) following the manufacturer’s protocol. Library size and uniformity were assessed using the Quantitative DNA HS kit on a TapeStation 4200 fragment analyzer (Agilent Technologies, Santa Clara, CA, USA).

Library concentrations were measured using a Qubit DNA HS assay kit (Thermo Fisher, Waltham, MA, USA). Equimolar library pools containing 48 samples were prepared and sequenced on the NextSeq 2000 platform (Illumina, San Diego, CA, USA) at a final pooled concentration of 650 pM using a single-end configuration (1 × 75 cycles).

The raw sequencing reads were processed and annotated using miRDeep2 against the reference genome GRCh38. Reads mapping to known and predicted microRNAs were quantified to generate raw count matrices for downstream analysis. The initial exploratory data visualization was performed with the objective of assessing the global structure of microRNA expression profiles across samples. Hierarchical clustering heatmaps were generated using row-scaled (z-score transformed) log2 counts per million (CPM) values to evaluate overall expression pattern similarities. Following an exploratory assessment, differential expression analysis was conducted using the edgeR package (v4.2.1) in R (v4.4.3). Lowly expressed miRNAs were filtered using the filterByExpr function. Library sizes were then normalized using the Trimmed Mean of M-values (TMM) method [38]. The estimation of dispersion parameters, encompassing both common and gene-wise (tagwise) dispersions, was accomplished utilizing the estimate Disp function. The differential expression between cases and controls was assessed using generalized linear models (GLMs) fitted with the quasi-likelihood framework (glmQLFit), followed by quasi-likelihood F-tests (glmQLFTest). *p*-values were adjusted for multiple testing using the Benjamini–Hochberg procedure in order to control the false discovery rate (FDR). MicroRNAs that exhibited a false discovery rate (FDR) less than 0.05 and a log2 fold change greater than 0.5 were considered to be significantly differentially expressed. Volcano plots were generated to visualize the relationship between effect size (log2 fold change) and statistical significance (−log10 FDR).

### 4.6. Selection of miRNAs Associated with Obesity and β-Cell Function to Be Validated by qRT–PCR

Metabolic pathways related to the expression of candidate miRNAs and the development of obesity (lipogenesis and insulin signaling) and pancreatic β-cell dysfunction (insulin synthesis and secretion, apoptosis) were identified using IPA software (QIAGEN Inc., Germantown, MD, USA, https://digitalinsights.qiagen.com/IPA, accessed on 30 March 2026) [39]. Target genes of interest were identified using the DIANA algorithm (microT-CDS) on the DIANA-TarBase v7.0 platform (https://www.ncbi.nlm.nih.gov/pubmed/25416803, accessed on 30 March 2026), which includes miRNAs reported in the miRNA database (miRBase, https://www.mirbase.org/, accessed on 30 March 2026) and described in the Kyoto Encyclopedia of Genes and Genomes (KEGG, https://www.genome.jp/kegg/, accessed on 30 March 2026).

### 4.7. Quantification of miRNAs by RT–qPCR

Real-time PCR was performed using TaqMan probes for miRNAs (Thermo Fisher, USA) following the manufacturer’s instructions. Synthetic has-miR-1249 (Thermo Fisher, USA. Id:002868) was used in plasma (10^−4^ pmol/μL) as a control, and the relative expression level was determined by the 2ΔCt method [40].

### 4.8. Expression of Target Genes of miRNAs by RT–qPCR

RNA extraction was performed using TRIZOL, and cDNA was synthesized with the M-MuLV-RT enzyme (New England Biolabs^®^, Ipswich, MA, USA). The primers for SOD (Forward: 5’TGG AAG TCG TTT GGC TTG 3’, Reverse: 5’CAG CTA GCA GGA TAA CAG ATG AG 3’) and GPx (Forward: 5’CAT CAG GAG AAC GCC AAG AA 3’, Reverse: 5’GCA CTT CTC GAA GAG CAT GA 3’) were designed at IDT (https://www.idtdna.com/, accessed on 30 March 2026) qPCR was performed using the SYBR Green detection system with 18S (Forward: 5’AAA CGG CTA CCA CAT CCA AG 3’, Reverse: 5’CCT CCA ATG GAT CCT CGT TA 3’) as constitutive genes. Relative gene expression was determined by the comparative double 2^−ΔΔCt^ method [40].

### 4.9. Statistical Analysis

Differential miRNA expression analysis was performed using the counts obtained from the annotation with the reference genome, and Fisher’s exact test was used to assess miRNA expression between groups. Heatmaps and volcano plots were created for data visualization. All the statistical analyses and data visualization for differential expressions were performed using R statistical software v. 4.4.3 (28 February 2025). Differences in continuous and categorical variables were assessed using Student’s *t*-test. The association between miRNAs and obesity was determined by logistic regression. The association between miRNAs and pancreatic β-cell function (insulin levels and the HOMA-β index) was determined using a linear regression model and Spearman’s rank correlation coefficient after its distribution was assessed with the Shapiro–Wilk test. SPSS software (version 22.0; IBM, Armonk, NY, USA) was used. Two-tailed *p* values <0.05 were considered significant. The sensitivity and specificity of miR-16-5p, miR-126-3p, and let-7a-5p for detecting cases and controls were assessed using the area under the ROC curve (AUC) and the 95% confidence interval (CI). To optimize the diagnostic capacity of microRNAs (miRNAs), clinical or laboratory variables that showed a significant Pearson correlation were integrated into the ROC curve analyses.

We used the STROBE reporting guideline to draft this manuscript, and the STROBE reporting checklist [41] when editing, included in Supplementary Materials (Supplementary Files S1).

## Figures and Tables

**Table 1:** Anthropometric parameters of the study population.

	Normal Weight	Obese
Sex (%)	40 G/60 B	40 G/60 B
Age (years)	9.02 ± 1.93	8.98 ± 1.76
Height (cm)	134.46 ± 13.18	138.9 ± 11.85
Weight (kg)	31.2 ± 9.2	49.64 ± 13.6 *
BMI (Percentile)	50.44 ± 23.25	97.54 ± 1.46 *
Waist circumference (cm)	60.62 ± 7.18	82.97 ± 9.48 *
Hip circumference (cm)	71.3 ± 8.74	89.36 ± 9.67
WHtR	0.46 ± 0.04	0.60 ± 0.06 *
Systolic/Diastolic BP (mm Hg)	97.74 ± 10.28/65.29 ± 6.28	98.6 ± 9.76/67.56 ± 6.63

G: girls; B: boys; BMI: body mass index; WHtR: waist-to-height ratio; BP: blood pressure. * *p* < 0.001; n = 50.

**Table 2:** Biochemical profile of the study population.

	Normal Weight	Obese
Glucose (mmol/L)	4.6 ± 0.44	4.82 ± 0.4
Insulin (µUI)	8.93 ± 5.59	18.5 ± 11.12 **
HOMA-IR	1.84 ± 1.16	4 ± 2.5 **
HOMA-β	168.4 ± 164	300.6 ± 179.7 **
TG (mg/dL)	144.6 ± 70.05	130.91 ± 52.7
TC (mg/dL)	162.02 ± 29.37	165.08 ± 28.54
HDL (mg/dL)	49.39 ± 19.91	45.53 ± 10.11
LDL (mg/dL)	83.71 ± 30.91	93.36 ± 24.73
TG/HDL-C ratio	2.51 ± 1.7,	3.10 ± 1.77 *
CMI	1.17 ± 0.84	1.87 ± 1.05 *

HOMA-IR: Homeostatic Model Assessment for Insulin Resistance; HOMA-β: Homeostasis Model Assessment of Beta-cell function; TG: triglycerides; TC: total cholesterol; HDL: high-density lipoproteins, LDL: low-density lipoproteins; CMI: estimated Cardiometabolic Index. ** *p* < 0.001 and * *p* < 0.01; n = 50.

## Data Availability

The original contributions presented in this study are included in the article/Supplementary Material. Further inquiries can be directed to the corresponding author.

## Supplementary Materials

The following supporting information can be downloaded at https://www.mdpi.com/article/10.3390/ijms27083396/s1.

## Abbreviations

The following abbreviations are used in this manuscript:

Akt	Protein kinase B
AUC	Area under the curve
B	Boys
BP	Blood pressure
C	Control group
CMI	Cardiometabolic index
G	Girls
GPx	Glutathione peroxidase
GSH	Reduced glutathione
HDL	High-density lipoprotein
HDL-C	High-density lipoprotein cholesterol
HbA1c	Glycated hemoglobin
HOMA-B	Homeostatic Model Assessment of B-cell function
HOMA-IR	Homeostatic Model Assessment for Insulin Resistance
IMSS	Instituto Mexicano del Seguro Social
IPA	Ingenuity Pathway Analysis
IR	Insulin resistance
KEGG	Kyoto Encyclopedia of Genes and Genomes
LDL-C	Low-density lipoprotein cholesterol
MAPK	Mitogen-Activated Protein Kinase
NGS	Next-generation sequencing
Ob	Obesity group
pBMI	Percentile Body mass index
PI3K	Phosphoinositide 3-kinase
ROC	Receiver Operating Characteristic
T2D	Type 2 diabetes
TC	Total cholesterol
TG	Triacylglycerols
VLDL	Very low-density lipoprotein cholesterol
WH	Circumference waist
WHtR	Waist-to-height ratio
