# Supplementary material for: Differential Expression of microRNAs in Obese Mexican Children: Links to Insulin Resistance and Dyslipidemia
Source: Int J Mol Sci. 2026 Apr 10;27(8):3396. doi: 10.3390/ijms27083396 (PMC13116648; doi:10.3390/ijms27083396)
Supplement: Supplementary file 1 [file ijms-27-03396-s001.zip › ijms-4215652-supplementary.pdf]

The STROBE reporting checklist

For checking that observational epidemiology research articles can be understood and used by everyone

	Item Description	Location (or reason for not reporting)
**Title and abstract**		
1a. Indicate the study's design	Indicate the study's design with a commonly used term in the title or the abstract.	Pag 1, abstract line 28
1b. Abstract	Provide in the abstract an informative and balanced summary of what was done and what was found.	Pag 1, abstract, line 30-35<br>Pag 1, abstract line 36-40
**Introduction**		
2. Background / rationale	Explain the scientific background and rationale for the investigation being reported.	Pag 2, introduction line 48-81
3. Objectives	State specific objectives, including any prespecified hypotheses.	Pag 2 introduction line 82-87
**Methods**		
4. Study design	Present key elements of study design early in the paper.	Pag 15, Sample Collection line 373-375
5. Setting	Describe the setting, locations, and relevant dates, including periods of recruitment, exposure, follow-up, and data collection.	Pag 15, Sample Collection line 373-375
6a. Eligibility criteria	**Cross-sectional study:** Give the eligibility criteria, and the sources and methods of selection of participants.	Pag 15, Sample Collection line 373-375
6b. Matching criteria	**Cohort study:** For matched studies, give matching criteria and number of exposed and unexposed.<br>**Case-control study:** For matched studies, give matching criteria and the number of controls per case.	Not applicable
7. Variables	Clearly define all outcomes, exposures, predictors, potential confounders, and effect modifiers. Give diagnostic criteria, if applicable.	Not applicable
8. Data sources / measurement	For each variable of interest give sources of data and details of methods of assessment (measurement). Describe comparability of assessment methods if there is more than one group.	Pag 15-17, Methods line 371-482
9. Bias	Describe any efforts to address potential sources of bias.	Pag 17, Study Limitations: line 364-369

10. Study size	Explain how the study size was arrived at.	Pag 15, Sample Collection line 373-374
11. Quantitative variables	Explain how quantitative variables were handled in the analyses. If applicable, describe which groupings were chosen, and why.	Pag 17, Differences in quantitative and categorical variables were assessed using Student's t tests. Pag 468-469.
12a. Statistical methods	Describe all statistical methods, including those used to control for confounding.	Pag 17, Statistical analysis. Line 464- 469
12b. Statistical methods – subgroups and interactions	Describe any methods used to examine subgroups and interactions.	Pag 17, Statistical analysis. Line 464-469
12c. Statistical methods – missing data	Explain how missing data were addressed.	Pag 17, Statistical analysis. Line 464-469
12di. Statistical methods – loss to follow-up	**Cohort study:** If applicable, describe how loss to follow-up was addressed.	Not applicable
12dii. Statistical methods – matching cases and controls	**Case-control study:** If applicable, explain how matching of cases and controls was addressed.	Not applicable
12diii. Statistical methods – sampling strategy	**Cross-sectional study:** If applicable, describe analytical methods taking account of sampling strategy.	Not applicable
12e. Statistical methods – sensitivity analyses	Describe any sensitivity analyses.	Pag 17, Statistical analysis. Line 464-469
**Results**		
13a. Participant numbers	Report the numbers of individuals at each stage of the study—e.g., numbers potentially eligible, examined for eligibility, confirmed eligible, included in the study, completing follow-up, and analysed; Consider use of a flow diagram.	Pag 15, **Sample Collection:** A total of 100 children. Line 373
13b. Participants – non-participation	Give reasons for non-participation at each stage.	Not applicable
13c. Participants – flow diagram	Consider use of a flow diagram.	Not applicable
14a. Descriptive data – participant characteristics	Give characteristics of study participants (e.g., demographic, clinical, social) and information on exposures and potential confounders. Present the information in a table.	Table 1 and table 2<br>Page: 3
14b. Descriptive data – missing data	Indicate the number of participants with missing data for each variable of interest.	Not applicable

14c. Descriptive data – follow-up time	**Cohort study:** Summarise follow-up time—e.g., average and total amount.	Not applicable
15. Outcome data	**Cohort study:** Report numbers of outcome events or summary measures over time. **Case-control study:** Report numbers in each exposure category, or summary measures of exposure. **Cross-sectional study:** Report numbers of outcome events or summary measures.	Not applicable
16a. Main results	Give unadjusted estimates and, if applicable, confounder-adjusted estimates and their precision (e.g., 95% confidence intervals). Make clear which confounders were adjusted for and why they were included.	The expression of the miRs (miR-16-5p, miR-126-3p and Let-7a) was used and adjusted with the correlation variable with 95% CI determined by Pearson or Spearman
16b. Main results – category boundaries	Report category boundaries when continuous variables were categorised.	Not applicable
16c. Main results – risk	If relevant, consider translating estimates of relative risk into absolute risk for a meaningful time period.	Not applicable
17. Other analyses	Report other analyses done—e.g., analyses of subgroups and interactions, and sensitivity analyses.	Pag 7-12, Results: line 173-246
**Discussion**		
18. Key results	Summarise key results with reference to study objectives.	Pag 15, discussion: line 358-361
19. Limitations	Discuss limitations of the study, taking into account sources of potential bias or imprecision. Discuss both direction and magnitude of any potential bias.	Pag 15, limitations: line 365-369
20. Interpretation	Give a cautious overall interpretation considering objectives, limitations, multiplicity of analyses, results from similar studies, and other relevant evidence.	Pag 12-15, discussion: line 249-362
21. Generalisability	Discuss the generalisability (external validity) of the study results.	Pag 12-15, discussion: line 249-362
**Other information**		
22. Funding	Give the source of funding and the role of the funders for the present study and, if applicable, for the original study on which the present article is based.	Pag 18, Funding: line 490-492
